# Co-design and evaluation of a digital serious game to promote public awareness about pancreatic cancer

**DOI:** 10.1186/s12889-024-18050-7

**Published:** 2024-02-22

**Authors:** Tara Anderson, Gillian Prue, Glenn McDowell, Patrick Stark, Christine Brown Wilson, Lisa Graham Wisener, Helen Kerr, Gemma Caughers, Katherine Rogers, Lana Cook, Stephanie Craig, Abdulelah Alanazi, Gary Mitchell

**Affiliations:** 1 School of Nursing and Midwifery, Queen’s University Belfast, Belfast, UK; 2 School of Psychology, Queen’s University Belfast, Belfast, UK; 3 Faculty of Applied Medical Sciences, Department of Nursing, The University of Bisha, Bisha, Saudi Arabia

**Keywords:** Pancreatic cancer, Co-design, Public awareness, Education, Serious game, Gamification, Public health

## Abstract

**Background:**

Pancreatic cancer, ranking seventh in global cancer-related deaths, poses a significant public health challenge with increasing incidence and mortality. Most cases are diagnosed at an advanced stage, resulting in low survival rates. Early diagnosis significantly impacts prognosis, making symptom awareness crucial. Symptoms are often subtle, leading to delayed help-seeking behaviour. Patients and their carers prioritise increased public awareness, indicating a need for innovative approaches to promote awareness of the disease.

**Methods:**

This study employed a quasi-experimental pre-test/post-test design to assess the relationship between a serious game and pancreatic cancer awareness. Members of the public (*N* = 727) were recruited internationally, via social media and with signposting by relevant organisations. Participants completed measures of symptom awareness and help-seeking intentions before and after playing the game. The serious game, co-designed with experts by lived experience, patient advocates and healthcare professionals, presented participants with a human anatomy diagram, with each section linked to a question about pancreatic cancer.

**Results:**

The serious game demonstrated a statistically significant improvement on pancreatic cancer awareness based on matched paired t-tests. Due to missing data, paired comparisons were only possible for 489 cases. Symptom awareness scores exhibited a statistically significant increase from pre-test to post-test, with a large effect size (*p* < 0.001, d = 1.43). Help-seeking intentions also markedly improved, showing a significant increase from pre-test to post-test, with a large effect size (*p* < 0.001, d = 1.10). Independent-samples t-tests were also conducted to determine if there were any group differences on pre- to post-test changes based on age, gender, and previous knowledge and/or experience of pancreatic cancer. Participants overwhelmingly endorsed the game’s usability and educational value, suggesting its potential as an effective tool for enhancing public awareness and proactive health-seeking behaviour.

**Discussion:**

This study is the first to explore a serious game’s utility in pancreatic cancer awareness. Results suggest that such interventions can effectively increase public awareness and influence help-seeking intentions. The co-design process ensured content relevance, and participant satisfaction was high. Findings highlight the game’s potential as an accessible and convenient tool for diverse populations.

**Supplementary Information:**

The online version contains supplementary material available at 10.1186/s12889-024-18050-7.

## Background

Pancreatic cancer is the seventh leading cause of cancer death globally [1]. Worldwide incidence and mortality have increased from 1990, and due to a lack of effective prevention and treatment strategies, pancreatic cancer represents a serious public health concern [2]. In the UK, pancreatic cancer is the 10th most common cancer with approximately 10,500 people diagnosed each year [3]. Approximately 80% of patients present with inoperable tumours at the time of their diagnosis and are therefore not eligible for potentially curative treatment [4]. As the majority of patients present symptomatic at an advanced stage [5], this has significant impact on symptom burden and life expectancy. In the UK, only 7% of people with pancreatic cancer survive for five years and survival rates have not improved much in the last forty years [3]. Earlier diagnosis has significant impact on prognosis; for example, 51% of those diagnosed at stage I reported 1-year survival compared to only 6% of those diagnosed at stage IV according to a recent Northern Ireland (NI) audit [5].

Symptoms of pancreatic cancer include jaundice, indigestion, change in bowel habits, fatigue, and unexplained weight loss [6]. Patients with pancreatic cancer may experience severe symptoms and poor quality of life to the end of life [7]. Patients have reported physical and psychological distress with pancreatic cancer patients reporting worse quality of life compared to other cancers [8–10]. Despite this high symptom burden and mortality rate, public awareness of pancreatic cancer is low. 64% of Europeans reported knowing very little about the disease [11]. Furthermore, individuals identified as high-risk (such as those with familial pancreatic cancer) have low levels of knowledge of pancreatic screening despite a desire to know more [12].

The subtle and intermittent nature of the symptoms of pancreatic cancer may cause delays in help-seeking with many individuals initially monitoring the symptoms and altering their diet [13]. Patients reported only visiting their General Practitioner (GP) after changes in symptom frequency, duration, or severity, with encouragement from friends and family facilitating help-seeking [13]. People living with pancreatic cancer have described the intermittent nature of their symptoms as creating a false sense of reassurance [14]. Due to the importance of earlier diagnosis on pancreatic cancer prognosis [4], this delayed help-seeking behaviour highlights the importance of addressing symptom awareness.

People living with pancreatic cancer and their carers have identified increasing public awareness of the disease symptoms as a research priority [15]. Raising symptom awareness coupled with improving self-efficacy for acknowledging and seeking help based on these symptoms may help to increase rates of earlier detection. Serious games represents a novel and innovative method of addressing such factors, this is because serious games are suggested to be more effective in terms of learning and knowledge retention than conventional instructions [16]. ‘Serious games’ (entertaining games created with a specific educational purpose) have become increasingly used within healthcare education [17] and show potential within public awareness. For example engagement with a serious game has shown improvements in the public’s perceptions of dementia [18] and increased awareness of the symptoms of Alzheimer’s Disease [19]. Serious games have also been found to address behaviour change in cancer patients, for example increases were found in self-advocacy [20], drug compliance [21] and engagement in preventative behaviours [22]. In the specific context of healthcare student education, serious games have elicited increases in nursing students’ knowledge and intentions to become vaccinated against influenza [23] and knowledge of infection and safe behaviours regarding COVID-19 [24].

The present study aims to extend this previous research to evaluate the effectiveness of a ‘serious game’ in increasing awareness of the symptoms of pancreatic cancer and help-seeking intentions within the general public. The objectives of this study were as follows:

1. To determine if playing a pancreatic cancer awareness game improved participants levels of awareness of the symptoms of pancreatic cancer.
2. To determine if playing a pancreatic cancer awareness game improved participants levels of help-seeking intentions.
3. To evaluate the acceptability of a pancreatic cancer awareness game to the general public as an educational tool to promote cancer awareness.

## Methods

### Design/ setting/ population

A quasi-experimental pre-test/post-test design was used to compare public awareness of the symptoms of pancreatic cancer before and after playing the digital, pancreatic cancer awareness, serious game. The questionnaires measured awareness of pancreatic cancer symptoms and help-seeking intentions. These were delivered immediately prior to and following participation in the game. The study was conducted using convenience sampling of members of the public with recruitment supported by the Northern Ireland Pancreatic Cancer Charity (NIPANC) and Queen’s University Belfast (QUB) social media channels during World Pancreatic Cancer Awareness month in November 2022.

### Co-design of intervention

The development of the digital serious game for pancreatic cancer involved a comprehensive co-design process in collaboration with 22 experts from Northern Ireland, representing a diverse array of perspectives including individuals affected by pancreatic cancer, advocates from cancer charities, relevant medical and healthcare professionals, and members of the public who served as end-users for the game.

The co-design process began with the formation of a collaborative team of experts, fostering a multidisciplinary approach. Subsequent co-design workshops engaged team members actively in the conceptualisation and design process, integrating their valuable insights into the serious game. The iterative design process allowed for multiple revisions, incorporating continuous feedback and refinements in collaboration with the co-design team to address specific insights and ensure alignment with educational goals and user experience. The interactive gameplay design presented players with a diagram of the human anatomy split into 15 tiles. Players selected tiles, answered questions, and progressed based on correct responses, fostering engagement and facilitating learning. Continuous feedback loops were established to provide insights on game mechanics, question content, and overall user experience, shaping the final product. Technical considerations were paramount in ensuring accessibility, and the game was developed as an HTML5 web application to guarantee usability on any device with a web browser.

Post-game, users were presented with a list of the most common symptoms of pancreatic cancer, reinforcing the educational aspect of the serious game. The co-design team collaborated with Focus Games Ltd to finalise the game, ensuring it effectively conveyed the desired educational content. The completed serious game is hosted online, allowing free access for users. The game can be freely accessed here: https://www.whatispancreaticcancer.co.uk/.

### Survey instrument

The pre- and post-measures were embedded within the game site, these can be viewed in Supplementary File 1. The pre-questionnaire recorded demographic details (gender, age, country, ethnic group, and knowledge and/or experience of cancer). This was followed by a measure of awareness of pancreatic cancer symptoms and help-seeking intentions adapted from a validated scale for self-efficacy around cancer symptoms [25]. This scale was adapted by the research team to be specific to pancreatic cancer and then piloted on 12 members of the public prior to administration. The first 20 items (α = 0.73) measured awareness of pancreatic cancer symptoms (e.g., yellowing of skin). Each of these items consisted of a five-point Likert scale ranging from very unlikely (0) to very likely [4]. The following seven items (α = 0.81) measured help-seeking intentions (e.g., I am able to pay attention to the symptoms of pancreatic cancer). The items obtained responses based on four-point Likert scale ranging from not true at all (0) to exactly true [3].

The post-questionnaire repeated the measures of awareness of pancreatic cancer symptoms and help-seeking intentions and included an evaluation of the game. This included a 14-item validated questionnaire adapted from Brooke (1996) [26] known as the ‘System Usability Scale’ (SUS) (α = 0.53). The items of this scale (e.g., I felt very confident using the game) consisted of a five-point Likert scale ranging from strongly disagree [1] to strongly agree [5].

### Data collection

Data collection for this study took place throughout November 2022 to mark World Pancreatic Cancer Awareness month. The general public could participate in this project between midnight on 01.11.2022 until midnight on 01.12.2022. Participants followed a link to the website where they could complete the study. A participant information sheet was embedded within the game outlining the details of the study. Participants were required to click a box to confirm they had read the information and consented to participate in the study. If participants did not consent to participate, they could still play the game without participating in the research study. All participants were permitted to withdraw from the study at any stage without giving any reason and information about these processes was provided within the participant information sheet.

The pre- and post-test measures were embedded within the game and completed before and after playing the game. Participants provided their email address for the purposes of linking the pre- and post-questionnaires.

### Ethics

Queen’s University Belfast, Faculty of Medicine, Health and Life Sciences Research Ethics Committee granted ethical approval for this study (Ref: MHLS22_145) after considering benefits and risks and ensuring participants autonomy would be respected. All methods were performed in accordance with the Declaration of Helsinki [27].

### Analysis

The pre- and post-test datasets were matched prior to analysis using email addresses which served as an identifier for each participant between the two datasets. All analyses were conducted in SPSS version 28.

Total scores for each questionnaire sub-scale were calculated based on Likert scale responses for both the pre- and post-test questionnaires. Several items were reverse coded to ensure higher scores reflected more accurate identification of symptoms or greater help-seeking intentions. Descriptive statistics were performed to observe demographic details of the participant sample. A series of paired t-tests were conducted to examine the change from pre-test to post-test for both the symptom awareness and help-seeking intentions subscales. Cohen’s d was calculated as a measure of effect size by dividing the difference between the pre- and post-test means by the pooled standard deviation. An independent-samples paired t-test was also conducted to examine any differences between those with no previous knowledge and/or experience of pancreatic cancer compared to those who had indicated some knowledge and/or experience. Further independent-samples t-tests were conducted to examine any group differences based on gender and age. Responses to the SUS at the post-test time point were analysed descriptively as percentages of response categories for each of the five Likert responses (strongly disagree to strongly agree).

In total, eight comparison analyses were conducted in this study. Therefore, a Bonferroni correction was applied to the alpha value when determining the statistical significance of results of these analyses to reduce the risk of false positives associated with multiple comparisons [28]. Alpha (0.05) was divided by the total number of comparisons [8] to give an alpha value of α = 0.00625. Results were therefore only considered to be statistically significant if their associated p-value was 0.00625 or below.

## Results

In total, 727 participants were recruited to evaluate the relationship of a serious game on awareness of the symptoms of pancreatic cancer and help-seeking intentions as assessed by pre- and post-questionnaires.

### Missing data

Primary analysis was possible for *N* = 489 cases out of a total of *N* = 727 due to missing data. If a participant was missing either pre-test or post-test data, they were excluded from paired t-test analyses, but their non-missing data was included in descriptive statistics. Missing data occurred due to a participant not completing either the pre- or post-test measures, or due to participants not supplying a correct identifier (email address) to allow their pre- and post-test data to be matched. Table 1 shows the level of missing data at pre-test, post-test, and analysis.

**Table 1:** Missing data

	Missing n (%)
Pre-test data	37 (5.1%)
Post-test data	201 (27.6%)
Paired comparisons	238 (32.7%)

### Demographic details

Due to substantial missing pre-test data and to ensure the demographic details presented reflect those included in the pre- and post-test analysis, participants with missing data have been excluded from the demographic details. Tables 2 and 3, therefore, provides participant demographics of those included in paired t-test analysis (*N* = 489). Most participants were from Northern Ireland (96.1%), white (96.3%), and female (92.0%). Age ranged from 16 to over 65 with the 18–25 age group representing the majority (71.8%).

**Table 2:** Participant descriptive statistics

		N	%
Gender	Female	450	92.0%
	Male	34	7.0%
	Non-Binary	2	0.4%
	Prefer Not to Say	3	0.6%
Age (years)	16–18	3	0.6%
	18–25	351	71.8%
	26–35	60	12.3%
	36–45	34	7.0%
	46–55	15	3.1%
	55–64	18	3.7%
	Over 65	8	1.6%
Ethnic Group	White	471	96.3%
	Mixed or Multiple Ethnic Groups	7	1.4%
	Black, Black British, Caribbean or African	5	1.0%
	Asian or Asian British	3	0.6%
	Arab	2	0.4%
	Kazakh	1	0.2%
Country	Northern Ireland	470	96.1%
	England	7	1.4%
	Republic of Ireland	9	1.8%
	Scotland	2	0.4%
	Netherlands	1	0.2%

Participants were also asked to indicate any previous knowledge and/or experience of cancer personally and/or professionally. Participants could select as many options as applied to them. Most participants were student health professionals (78.1%) while a substantial proportion of the sample had a family member who lives or lived with cancer (47.9%). Contrastingly, only a minority of the sample had a family member who lives or lived with pancreatic cancer (7.6%). Further descriptive statistics regarding participants’ experience is presented in Table 3.

**Table 3:** Participant knowledge and/or experience of cancer descriptive statistics

	N	%
I am a health professional	36	7.4%
I am a health professional that provides care to people with cancer	26	5.3%
I am a health professional that provides care to people with pancreatic cancer	6	1.2%
I am a student health professional (e.g., medicine, nursing, allied health)	384	78.1%
I am part of a charity, network or organisation that provides support to people affected by pancreatic cancer	5	1.0%
I have a family member who lives or lived with cancer	234	47.9%
I have a family member who lives or lived with pancreatic cancer	37	7.6%
I live or have lived with cancer	7	1.4%
I live or have lived with pancreatic cancer	3	0.6%
I have received training or education about pancreatic cancer in the past	7	1.4%
None of these apply to me	36	7.3%
Valid N (listwise)	489	

### Pre-test to post-test changes in symptom awareness and help-seeking intentions

#### Symptom awareness

Participants showed increased scores on the post-test measure of symptom awareness (*M* = 59.10, *SD* = 8.31) compared to their pre-test score (*M* = 47.08, *SD* = 6.87), as shown in Table 4. A paired samples t-test was used to determine whether there was a statistically significant mean difference between pre- and post-test symptom awareness scores for the sample of *N* = 489 who completed both the pre-test and post-test. This found a statistically significant mean increase of *M* = 13.41, 95% CI [14.24, 12.58], *t*(488) = 31.64, *p* < 0.001, with large effect size (*d* = 1.43).

**Table 4:** Descriptive statistics for pre-test and post-test total symptom awareness scores

	N	Range	Minimum	Maximum	Mean	Std. Deviation
Symptom awareness pre-test	690	53	21	74	47.08	6.87
Symptom awareness post-test	526	53	27	80	59.10	8.31
Valid N (listwise)	489					

Descriptive statistics are also presented for individual symptoms present in the symptom awareness questionnaire. Items were coded so that higher scores reflect more accurate responses. For example, a higher score for a correct symptom reflects increased identification of this symptom by participants. Whereas a higher score for an incorrect symptom reflects decreased identification of this symptom by participants. Table 5 presents the items presented to participants. The colour green represents actual symptoms of pancreatic cancer, and the colour yellow represents symptoms that are not commonly associated with pancreatic cancer.

**Table 5:** Descriptive statistics for pre-test and post-test individual symptoms

Symptom	Pre-test		Post-test	
	Mean	Std. Deviation	Mean	Std. Deviation
Yellowing of Skin*	2.96	0.84	3.58	0.75
Yellowing of Eyes*	2.90	0.86	3.37	0.93
Fatigue*	3.21	0.80	3.65	0.65
Blurred Vision	1.79	0.80	2.17	1.10
Lower Back Pain	1.38	0.87	2.05	1.30
Middle Back Pain*	2.67	0.80	3.41	0.85
Pale Poo*	2.52	0.87	3.33	1.02
Dark Poo	2.04	0.86	2.72	1.20
Smelly Poo*	2.46	0.81	3.07	1.11
Blood in Poo	1.58	0.92	2.12	1.13
Pins & Needles or Numbness	1.75	0.79	2.32	1.03
Diabetes*	2.48	0.94	3.29	0.88
Indigestion*	2.63	0.87	3.61	0.67
Unexplained Weight Loss*	3.14	0.80	3.60	0.69
Low Mood*	2.75	0.88	3.60	0.69
Pain on Eating*	2.62	0.84	3.44	0.84
Lower Abdominal Pain	1.37	0.88	1.83	1.28
Upper Abdominal Pain*	2.57	0.83	3.40	0.86
Itchy Skin*	2.41	0.88	2.23	1.20
Leg Swelling	1.84	0.81	2.31	1.05
Valid (N) listwise	*N* = 690		*N* = 526	

*Correct symptom

#### Help-seeking intentions

Participants showed increased scores on the post-test measure of help-seeking intentions (*M* = 17.03, *SD* = 3.13) compared to their pre-test score (*M* = 12.83, *SD* = 4.12), as shown in Table 6. A paired samples t-test was used to determine whether there was a statistically significant mean difference between pre- and post-test help-seeking intentions scores for the sample of *N* = 489 who completed both pre- and post-test measures. This found a statistically significant mean increase of *M* = 4.51, 95% CI [4.88, 4.15], *t*(488) = 24.25, *p* < 0.001, with large effect size (*d* = 1.10).

**Table 6:** Descriptive statistics for pre-test and post-test total help-seeking intentions scores

	N	Range	Minimum	Maximum	Mean	Std. Deviation
*Help-seeking intentions pre-test*	690	21	0	21	12.83	4.12
*Help-seeking intentions* post-test	526	21	7	21	17.03	3.13
Valid N (listwise)	489					

### Group differences based on previous experience

Although most participants (*N* = 453) indicated that they had previous knowledge and/or experience of pancreatic cancer, 36 participants indicated that none of the given categories applied to them. Further descriptive statistics for previous knowledge and/or experience are presented above in Table 3. Two independent-samples t-tests were conducted to determine if there were differences between these groups on pre- to post-test increases in both symptom awareness and help-seeking. No statistically significant group differences were found.

In addition, independent-samples t-tests were conducted to determine if there were any group differences based on the majority age group (18–25; *N* = 351) and gender (female; *N* = 450) compared to the rest of the sample. These were conducted for pre- to post-test increases for both symptom awareness and help-seeking intentions. Table 7 presents the means, standard deviations, 95% confidence intervals (CIs), and *p* values for these tests.

**Table 7:** Results of the independent-samples t-tests

	Symptom awareness		Help-seeking intentions	
	Mean (Std. deviation)	95% CIs (p value)	Mean (Std. deviation)	95% CIs (p value)
Previous knowledge and/or experience	13.31 (9.39)	-4.58 to 1.80 (*p* = 0.39)	4.45 (4.04)	-2.54 to 0.89 (*p* = 0.34)
No previous knowledge and/or experience	14.69 (9.12)		5.28 (4.97)	
Female	13.51 (9.26)	-1.77 to 4.38 (*p* = 0.40)	4.57 (4.03)	-0.57 to 2.13 (*p* = 0.26)
Other gender	12.21 (10.60)		3.79 (4.95)	
Aged 18–25	13.86 (9.23)	-3.46 to 0.24 (*p* = 0.09)	4.17 (4.15)	-1.29 to 0.33 (*p* = 0.25)
Other age groups	12.25 (9.66)		4.65 (4.10)	

### Game evaluation

526 participants completed the game evaluation present on the post-questionnaire. Responses to the System Usability Scale (SUS) are shown below in Table 8. Overall, there was a clear majority positive response (either agree or strongly agree) to all positively scored items, e.g. “I think that I would like to use this game frequently”. While there was a clear majority negative response (either disagree or strongly disagree) to all negatively scored items, e.g. “I found the game unnecessarily complex”.

**Table 8:** Descriptive statistics for system usability scale

	Strongly disagree (%)	Disagree (%)	Neutral (%)	Agree (%)	Strongly agree (%)
I think that I would like to use this game frequently	1.0%	2.9%	12.4%	37.4%	18.7%
I found the game unnecessarily complex*	27.4%	34.5%	6.2%	2.6%	1.7%
I thought the game was easy to use	0.6%	1.7%	2.9%	29.8%	37.4%
I think that I would need the support of a technical person to be able to use this game*	47.5%	19.8%	2.8%	1.8%	0.6%
I found the various functions in this game were well integrated	0.7%	1.2%	6.1%	38.8%	25.6%
I thought there was too much inconsistency in this game*	32.9%	33.0%	4.1%	1.8%	0.6%
I would imagine that most people would learn to use this game very quickly	0.3%	1.1%	2.6%	29.6%	38.8%
I found the game very cumbersome to use*	22.7%	22.4%	16.9%	6.1%	4.3%
I felt very confident using the game	0.7%	1.0%	5.1%	32.9%	32.7%
I needed to learn a lot of things before I could get going with this game*	22.0%	29.0%	9.5%	9.1%	2.8%
I found this game educational	0.4%	0.1%	1.9%	26.0%	43.9%
I would recommend this game to other people	0.3%	1.5%	4.1%	28.7%	32.7%
I enjoyed playing this game	0.4%	0.4%	4.8%	32.0%	34.7%
I will play this game more than once	1.8%	5.9%	14.7%	27.1%	22.8%

*Negatively scored items

A scale-score was also computed for the System Usability Scale and negatively scored items were reverse coded to ensure higher scores reflected more positive responses. A mean score of 59.16 out of a possible 70 was found. Further descriptive data is presented in Table 9.

**Table 9:** Descriptive statistics for total SUS scores

	N	Range	Minimum	Maximum	Mean	Std. Deviation
Total SUS Score	526	56	14	70	59.16	7.19

Additionally, participants were asked to rate the game on a scale with five stars indicating an excellent review and one star indicating a poor review. Most participants provided either a 4-star (21.7%) or 5-star (45.9%) rating. Further descriptive statistics are presented in Table 10.

**Table 10:** Descriptive statistics for game rating

	1	2	3	4	5
Please provide a rating for the game based on your enjoyment and learning (higher star denotes higher satisfaction).	0.1%	0.3%	4.3%	21.7%	45.9%

### Summary of results

To summarise, 727 members of the public were recruited to evaluate the relationship between a pancreatic cancer awareness game and symptom awareness and help-seeking intentions. Due to missing data, pre and post-test analysis was possible for *N* = 489 cases. Findings demonstrate statistically significant (*p* < 0.001) mean increases in both symptom awareness scores and help-seeking intentions from pre-test to post-test levels. Both of these increases also showed large effect size. No group differences were found on pre- to post-test increases in either symptom awareness or help-seeking intentions for previous knowledge and/or experience, gender, or age. Additionally, game evaluation data revealed a predominantly favourable response.

## Discussion

Pancreatic cancer is a growing global public health concern largely due to the subtlety of symptoms [1, 2]. Improving public awareness of the symptoms of pancreatic cancer as well as self-efficacy for seeking help based on these may help to facilitate earlier detection which has significant impact on prognosis [5]. To the best of our knowledge, this is the first study to investigate the utility of a ‘serious game’ within this context. Following engagement with the game, participants developed a statistically significantly greater level of symptom awareness and increased help-seeking intentions scores. These improvements were demonstrated with large effect size, highlighting the potential of this pancreatic cancer awareness game as an educational tool. Further, the study found that, despite variations in participants’ prior knowledge and experience with pancreatic cancer, there were no significant differences in increased symptom awareness or help-seeking intentions between different groups, including those categorised by age and gender. The lack of significant differences in increased symptom awareness and help-seeking intentions across various participant groups suggests that the awareness intervention had a consistent impact, which can be viewed as a positive outcome, indicating that the educational efforts appear to show the same relationship across diverse demographics.

Participants showed increased levels of symptom awareness supporting previous research that a serious game can elicit an increase in knowledge [16, 18, 20, 27, 28]. The present study extends these findings to the context of pancreatic cancer symptoms, suggesting this serious game may be beneficial in increasing public awareness of the symptoms of pancreatic cancer which is currently lacking [11, 12]. However, it is important to note that participants may have had prior knowledge of these symptoms as a substantial proportion were student healthcare professionals and/or had personal experience of a family member with cancer. Therefore, findings may reflect increases from a baseline level greater than that of the general public.

The additional finding of increases in self-reported help-seeking intentions following engagement with the game is also in line with previous research [20, 22, 25]. Improved symptom awareness coupled with increased help-seeking intentions (when experiencing identified symptoms) may help lead to earlier detection which has significant impacts on prognosis and survival rates [5]. This is especially encouraging as the nature of pancreatic cancer symptoms is often subtle and intermittent which has been reported to cause delays in help-seeking [13, 14]. Although these findings are promising given their emergence in a short time frame following engagement with the game, it is not possible to make conclusions on retention of this knowledge as post-test data was obtained directly after game play.

The content of the serious game was determined by a co-design group including individuals with expertise in pancreatic cancer, people living with pancreatic cancer, and members of the public. Increasing public awareness of the symptoms has previously been identified as a research priority by those living with the disease and their carers [15]. The co-design process utilised in the present study allowed the priorities of those with professional expertise, lived experience, and the end users (public) to be incorporated into the short game. Such games have shown promise in previous research [18]. Further, similar to previous research the present study found high levels of satisfaction with the serious game [20, 21, 24]. Most participants reported confidence and enjoyment in using the game and rated the game very highly. The large sample size recruited in the present study suggests that this serious game provides an accessible and convenient mode of learning for a wide population.

Serious games have become a powerful tool for public health promotion, especially among younger people globally [29]. Using serious games is gaining popularity in chronic disease self-management, offering a motivational approach with elements like points and badges [30]. Challenges persist in designing games that align with educational curricula, maintain engagement, and undergo rigorous evaluation [29–31]. While serious games show promise in addressing public health concerns, the field needs a multidisciplinary approach to ensure effectiveness and cultural relevance. Overcoming challenges in evaluation methods and ensuring standardised measures for engagement will be crucial for maximising the impact of serious games on health promotion across diverse age groups [32].

In comparison to traditional methods of spreading public health awareness, such as pamphlets, posters, and lectures, serious games offer a dynamic and interactive approach that resonates particularly well with the younger population as evidenced by this study (32–33). While traditional methods rely on passive consumption of information, serious games actively engage users, enhancing comprehension and knowledge retention (34–35). The potentially immersive and entertaining nature of serious games fosters a positive learning environment, making health information more accessible and appealing [36]. Additionally, serious games allow for the incorporation of behaviour change theories, providing a theoretical framework to drive positive health outcomes [29–32].

The findings of this study indicate that this serious game shows promise as an educational tool to promote public awareness of pancreatic cancer symptoms and improve intentions to seek help based on these symptoms. This is especially important within the context of pancreatic cancer where early detection and diagnosis can have such a substantial impact on prognosis [4]. Although the generalisability of this finding may be limited due to the level of knowledge and/or experience with cancer of the participant population. It is encouraging that the serious game evaluated in this study provides a novel approach to addressing this.

### Strengths and limitations

Social media recruitment proved successful in obtaining a large sample size for this study with participants aged between 16 and over 65 and respondents from five countries. However, most of the sample were from Northern Ireland, white, female, and aged 18–25. This may limit the generalisability of the findings to a more diverse population. Although the age range may suggest the younger population are more likely to engage with serious gaming, previous research has found serious games to be appealing to a diverse population regardless of age [37]. This age range may instead represent the student health professionals who made up most of the sample. The use of convenience sampling via social media therefore would make generalisability challenging to the whole population.

This substantial proportion of respondents who were student health professionals in addition to those who had a family member who lives or lived with cancer may present another limitation. Such participants may have had a greater level of symptom awareness prior to their engagement with the resource when compared to the general public. However, a minority of participants had a family member who lives or lived with pancreatic cancer specifically. This suggests this intervention may reach beyond those who are naturally more invested in the topic due to personal experience. Further comparative analysis between specific participant groups may be helpful to determine any group differences between those with prior knowledge and/or experience and with those without. However, analysis was limited with the current sample because the response options allowed participants to select multiple categories that were not mutually exclusive. For instance, participants could indicate both being a health professional and having specific experience with pancreatic cancer, leading to a complex and overlapping categorisation. This inherent ambiguity in participant responses made straightforward division of participants into distinct groups for meaningful comparative analysis complex.

The authors opted for the System Usability Scale (SUS) due to its simplicity and efficient administration, employing 10 Likert-type questions to enhance response rates. Notwithstanding its prior validation, previous research has identified low internal consistency, posing potential limitations on the interpretability of SUS results. This observed reduction in reliability warrants a cautious approach when drawing conclusions from the questionnaire within the scope of our research.

In terms of future research, it would be useful to extend the current findings by examining the long-term outcomes of engaging with the serious game. This could help to shed light on learning retention and any sustained impact of the increase in help-seeking intentions. Such sustained increases could benefit individuals who may encounter symptoms later in life. The present study also measured help-seeking intentions, future research investigating actual change in help-seeking behaviour would be beneficial. In addition, it may be helpful to compare the outcomes of this serious game with other interventions. Although the pre- and post-test design of the present study represents a pragmatic and cost-effective method of evaluating the intervention, this design does not have a comparison or control group. Therefore, future research would benefit from testing the effectiveness of the pancreatic cancer awareness game in a randomised control trial (RCT).

## Conclusion

The use of a serious digital game has resulted in a positive relationship on both pancreatic symptom awareness and help-seeking intentions. The game was also positively evaluated by participants. In summary, digital gaming is an accessible mode to reach a wide audience and may help to improve earlier detection of pancreatic cancer. However, future research is needed to reach a more diverse population and test the intervention within an RCT to provide more reliable evidence regarding its effectiveness.

### Electronic supplementary material

Below is the link to the electronic supplementary material.

Supplementary Material 1 - Pancreatic Cancer Awareness Game Pre/Post Questionnaire

## Data Availability

The datasets used and/or analysed during the current study are available from the corresponding author on reasonable request.

## References

[1] Bray F, Ferlay J, Soerjomataram I, Siegel RL, Torre LA, Jemal A (2018). Global cancer statistics 2018: GLOBOCAN estimates of incidence and mortality worldwide for 36 cancers in 185 countries. CA Cancer J Clin.

[2] Kan C, Liu N, Zhang K, Wu D, Liang Y, Cai W, et al. Global, Regional, and national burden of pancreatic cancer, 1990&ndash;2019: results from the global burden of disease study 2019. Ann Glob Health. 2023 May 25;89(1):33. 10.5334/aogh.4019 PMC10215993 37252335

[3] Cancer Research UK. Pancreatic Cancer [Internet]. 2023 [cited 2023 Oct 18]. Available from: https://www.cancerresearchuk.org/about-cancer/pancreatic-cancer.

[4] Partelli S, Sclafani F, Barbu ST, Beishon M, Bonomo P, Braz G (2021). European Cancer Organisation Essential Requirements for Quality Cancer Care (ERQCC): pancreatic Cancer. Cancer Treat Rev.

[5] Hawkins S, Santos R, Johnston D, McCain S, Bennett D, Coleman H. Northern Ireland Pancreatic Cancer Audit, Measuring the quality of care for patients diagnosed 2019–2020. NIreland Cancer Regist Queens Univ Belf [Internet]. 2023; Available from: https://pureadmin.qub.ac.uk/ws/portalfiles/portal/460628359/Filetoupload_1756444_en.pdf.

[6] NIPANC. Symptoms of Pancreatic Cancer [Internet]. 2023 [cited 2023 Oct 18]. Available from: https://www.nipanc.org/pancreatic-cancer.

[7] Lee V, Cheng H, Li G, Saif MW. Quality of Life in Patients with Pancreatic Cancer. 2012. 22406597

[8] Bauer MR, Bright EE, MacDonald JJ, Cleary EH, Hines OJ, Stanton AL (2018). Quality of life in patients with pancreatic Cancer and their caregivers: a systematic review. Pancreas.

[9] Chung V, Sun V, Ruel N, Smith TJ, Ferrell BR (2022). Improving Palliative Care and Quality of Life in Pancreatic Cancer patients. J Palliat Med.

[10] Clark KL, Loscalzo M, Trask PC, Zabora J, Philip EJ (2010). Psychological distress in patients with pancreatic cancer-an understudied group: psychological distress in patients. Psychooncology.

[11] Celgene Coporations in conjunction with Ipsos Public Affairs. PANCREATIC CANCER AWARENESS SURVEY GLOBAL ONLINE OMNIBUS SURVEY [Internet]. 2014 Nov. Available from: https://media.celgene.com/content/uploads/2014/11/pancreatic-cancer-awareness-survey.pdf.

[12] Lewis ZK, Frost CJ, Venne VL (2009). Pancreatic Cancer surveillance among high-risk populations: knowledge and intent. J Genet Couns.

[13] Mills K, Birt L, Emery JD, Hall N, Banks J, Johnson M (2017). Understanding symptom appraisal and help-seeking in people with symptoms suggestive of pancreatic cancer: a qualitative study. BMJ Open.

[14] Evans J, Chapple A, Salisbury H, Corrie P, Ziebland S (2014). It can’t be very important because it comes and goes—patients’ accounts of intermittent symptoms preceding a pancreatic cancer diagnosis: a qualitative study: table 1. BMJ Open.

[15] Saunders C, Gooden H, Robotin M, Mumford J (2009). As the bell tolls: a foundation study on pancreatic cancer consumer’s research priorities. BMC Res Notes.

[16] Wouters P, Van Nimwegen C, Van Oostendorp H, Van Der Spek ED (2013). A meta-analysis of the cognitive and motivational effects of serious games. J Educ Psychol.

[17] Zhonggen Y (2019). A Meta-analysis of Use of Serious games in Education over a Decade. Int J Comput Games Technol.

[18] Carter G, Brown Wilson C, Mitchell G. The effectiveness of a digital game to improve public perception of dementia: A pretest-posttest evaluation. Pan X, editor. PLOS ONE. 2021;16(10):e0257337. 10.1371/journal.pone.0257337 PMC8500403 34624040

[19] Cook B, Twidle P. Increasing Awareness of Alzheimer’s Disease through a Mobile Game. In: 2016 International Conference on Interactive Technologies and Games (ITAG) [Internet]. Notthingham, United Kingdom: IEEE; 2016 [cited 2023 Jul 14]. p. 55–60. Available from: http://ieeexplore.ieee.org/document/7782515/.

[20] Thomas TH, Bender C, Donovan HS, Murray PJ, Taylor S, Rosenzweig M et al. The feasibility, acceptability, and preliminary efficacy of a self-advocacy serious game for women with advanced breast or gynecologic cancer. Cancer. 2023;cncr.34887. 10.1002/cncr.34887 PMC12105837 37243943

[21] Kim HJ, Kim SM, Shin H, Jang JS, Kim YI, Han DH (2018). A Mobile game for patients with breast Cancer for Chemotherapy Self-Management and Quality-of-life improvement: Randomized Controlled Trial. J Med Internet Res.

[22] Wochna Loerzel V, Clochesy J, Geddie P (2020). Using Serious games to increase Prevention and Self-Management of Chemotherapy-Induced nausea and vomiting in older adults with Cancer. Oncol Nurs Forum.

[23] Mitchell G, Leonard L, Carter G, Santin O, Brown Wilson C. Evaluation of a ‘serious game’ on nursing student knowledge and uptake of influenza vaccination. Duplaga M, editor. PLOS ONE. 2021;16(1):e0245389. 10.1371/journal.pone.0245389 PMC7808644 33444348

[24] Calik A, Cakmak B, Kapucu S, Inkaya B (2022). The effectiveness of serious games designed for infection prevention and promotion of safe behaviors of senior nursing students during the COVID-19 pandemic. Am J Infect Control.

[25] De Nooijer J, Lechner L, Candel M, De Vries H (2004). Short- and long-term effects of tailored information versus general information on determinants and intentions related to early detection of cancer. Prev Med.

[26] Brooke J. SUS - A quick and dirty usability scale. 1996.

[27] World Medical Association (2001). World Medical Association Declaration of Helsinki. Ethical principles for medical research involving human subjects. Bull World Health Organ.

[28] Bland JM, Altman DG (1995). Multiple significance tests: the Bonferroni method. BMJ.

[29] Damaševiˇcius R, Maskeliunas R, ¯ Blažauskas T (2023). Serious games and Gamification in Healthcare: a Meta-review. Information.

[30] Huang X, Xiang X, Liu Y, Wang Z, Jiang Z, Huang L (2023). The Use of Gamification in the self-management of patients with chronic diseases: scoping review. JMIR Serious Games.

[31] Sharifzadeh N, Kharrazi H, Nazari E, Tabesh H, Edalati Khodabandeh M, Heidari S, Tara M (2020). Health Education Serious Games Targeting Health Care Providers, patients, and Public Health Users: scoping review. JMIR Serious Games.

[32] Andrew L, Barwood D, Boston J, Masek M, Bloomfield L, Devine A (2023). Serious games for health promotion in adolescents - a systematic scoping review. Educ Inform Technol.

[33] Crooks S, Carter G, Wilson CB, Wynne L, Stark P, Doumas M, Rodger M, O’Shea E, Mitchell G (2023). Exploring public perceptions and awareness of Parkinson’s disease: a scoping review. PLoS ONE.

[34] Craig S, Mitchell G, Halloran PO, Stark P, Wilson CB (2023). Exploring the experiences of people living with dementia in Dementia Friendly communities (DFCs) in Northern Ireland: a realist evaluation protocol. BMC Geriatr.

[35] Craig S, Stark P, Wilson CB (2023). Evaluation of a dementia awareness game for undergraduate nursing students in Northern Ireland: a Pre-/Post-Test study. BMC Nurs.

[36] Mitchell G, Carter G, Brown Wilson C (2021). Gaming for awareness. J Dement Care.

[37] DeSmet A, Van Ryckeghem D, Compernolle S, Baranowski T, Thompson D, Crombez G (2014). A meta-analysis of serious digital games for healthy lifestyle promotion. Prev Med.

