# Supplementary material for: Co-design and evaluation of a digital serious game to promote public awareness about pancreatic cancer
Source: BMC Public Health. 2024 Feb 22;24:570. doi: 10.1186/s12889-024-18050-7 (PMC10885377; doi:10.1186/s12889-024-18050-7)
Supplement: Supplementary file 1 — Supplementary Material 1 - Pancreatic Cancer Awareness Game Pre/Post Questionnaire [file 12889_2024_18050_MOESM1_ESM.doc]

**Supplementary File 1: Pancreatic Cancer Awareness Game Pre/Post Questionnaire**

**Identifying pancreatic cancer symptoms**

Please rate the likelihood of the below items to be a symptom of pancreatic cancer on the following scale: 1very unlikely, 2 unlikely, 3 unsure, 4 likely, 5 very likely. (*Denotes correct symptom).

Yellowing of Skin*

Yellowing of Eyes*

Fatigue*

Blurred Vision

Lower Back Pain

Middle Back Pain*

Pale Poo*

Dark Poo

Smelly Poo*

Blood in Poo

Pins & Needles or Numbness

Diabetes*

Indigestion*

Unexplained Weight Loss*

Low Mood*

Pain on Eating*

Lower Abdominal Pain

Upper Abdominal Pain*

Itchy Skin*

Leg Swelling

**Awareness and Help Seeking Intensions**

Please rate the below items for how true they are for you, on the following scale

1 not true at all, 2 hardly true, 3 moderately true, 4 exactly true

1. ‘I am able to pay attention to the symptoms of pancreatic cancer’
2. ‘I find it difficult to pay attention to symptoms of pancreatic cancer’
3. ‘I am able to seek help for pancreatic cancer symptoms’
4. ‘I am able to seek help when I doubt whether a symptom is a pancreatic cancer symptom’
5. ‘I am able to seek help when I expect the doctor to think the complaint is not serious’
6. ‘I am able to seek help when I expect the complaint to not be serious’
7. ‘I am able to seek help when I experience fear’
